# Using Design Thinking for Co-Creating an Integrated Care Pathway Including Hospital at Home for Older Adults with an Acute Moderate-Severe Respiratory Infection in the Netherlands

**DOI:** 10.5334/ijic.6991

**Published:** 2023-06-21

**Authors:** Rianne M. C. Pepping, Maarten O. van Aken, Rimke C. Vos, Mattijs E. Numans, Johanna M. W. van den Berg, Ingrid Kroon, Cees van Nieuwkoop

**Affiliations:** 1 Department of Public Health & Primary Care/Health Campus The Hague, Leiden University Medical Center, The Hague, The Netherlands; 2 Department of Internal Medicine, Haga Teaching Hospital, The Hague, The Netherlands; 3 Department of Pulmonology, Haga Teaching Hospital, The Hague, The Netherlands; 4 Elderly Care Medicine, Florence Health & Care, The Hague, The Netherlands

**Keywords:** Integrated Care Pathway, hospital at home, respiratory infection, older adults, patient journey, design thinking

## Abstract

**Introduction:**

Acute respiratory infections are common in frail, community-dwelling older people and are accompanied by considerable diagnostic and prognostic uncertainties. Inadequately coordinated care is associated with unnecessary hospital referral and admission with potential iatrogenic harm. Therefore, we aimed to co-create a regional integrated care pathway (ICP), including a hospital at home journey.

**Developing the ICP:**

Tasked with using design thinking methodology, stakeholders from regional healthcare facilities, together with patient representatives, were assigned to different focus groups based on their expertise. The focus of each session was to co-create ideal patient journeys suitable for embedding in the ICP.

**Results:**

Based on these sessions, a regional cross-domain ICP was developed that comprises three patient journeys. The first journey included a hospital at home track, the second a tailored visit, with priority assessment, to regional emergency departments, and the third concerned referral to readily available nursing home ‘recovery-beds’ under the supervision of an elderly care medicine specialist.

**Conclusion:**

Using design thinking and involving end-users during the whole process, we created an ICP for community-dwelling frail older people with moderate-severe acute respiratory infections. This resulted in three realistic patient journeys, including a hospital at home track, which will be implemented and evaluated in the near future.

## Introduction

Medical management of community-dwelling, presumably frail, older people (aged ≥ 65 years), with acute respiratory infections is challenging for both the general practitioner (GP) and the hospital specialist. Acute respiratory infections are frequently characterized by diagnostic and prognostic uncertainties attributable to overlapping symptoms and the often-unclear concurrent effects of comorbidities such as heart failure (HF) and chronic obstructive pulmonary disease (COPD) [1,2]. A referral, followed by an assessment at a hospital emergency department (ED), is strongly associated with an increased risk of functional decline [3,4,5]. The decision to either refer a frail patient to a hospital for diagnostics, admission and/or treatment or to keep the patients in their home environment is a difficult one because both choices carry health risks [6,7]. When the choice is hospitalization, risks of iatrogenic harm such as delirium or pressure ulcers due to a forced bedridden status are introduced [8,9,10,11,12]. The latter is associated with overall deconditioning and considered one of the main causes of poorer health outcomes post-discharge [13]. Hospitalization of frail older people should therefore be avoided whenever possible [14,15,16].

Nonetheless, frail older people diagnosed with moderate to severe pneumonia, for whom intensive care treatment is judged inappropriate or futile, are usually admitted to general wards and treated empirically with intravenous antibiotics according to current guidelines [17,18]. However, the general prognosis of moderate to severe pneumonia in frail older people assigned a non-intensive care treatment strategy, regardless of choice of antibiotic treatment, is similar whether in a hospital or home setting [19,20], and several studies have highlighted the safety and advantages of treatment in a ‘hospital at home’ setting [21]. Currently, the existing regional healthcare system in the Netherlands does not include hospital at home treatment options.

Respiratory infections are the most common infectious diseases associated with hospital admissions, with estimated costs of 280 million euros yearly in the Netherlands. The reported prevalence of GP-diagnosed respiratory infections was 16.3/1000 in 2017 [22]. With an aging population, respiratory infections are expected to increase by 27% in 2040 [22]. Of the associated hospital admissions, almost 75% involve patients of 65 years or older. The median length of stay (LOS) is six days, a period often extended with non-medical hospital days [23] due to, for example, delays when a patient has additional home care requirements or waiting lists for nursing home recovery beds, compounding the risks mentioned above.

In the Netherlands, current treatment protocols for pneumonia rarely include recommendations on care coordination between the care partners involved. In our urban region at least, this is not yet present. GPs, hospitals, nursing homes and home care organizations generally lack the resources, like staff and bed capacity, to deliver integrated care [24,25,26,27]. As a consequence, respiratory infections in older people often result in unnecessary – or unnecessarily lengthy – hospital admissions.

Due to demographic factors – such as the rise in chronic diseases and concomitant health care needs, as well as greater patient desire regarding personalized medicine – there is a need for an integrated care pathway (ICP) for disease management of respiratory diseases. An ICP, a ‘clinical pathway’ or a ‘care pathway’ are all terms that describe the same concept. This concept involves “structured multidisciplinary care plans which detail essential steps in the care of patients with a specific clinical problem. They support the translation of clinical guidelines into local protocols and clinical practice [28].” An ICP that includes a hospital at home treatment would likely contribute to better health outcomes for older people, have a beneficial impact on patient’s experience and health care resources and costs [28,29].

The aim of this study was to develop a regional, multidisciplinary ICP with multiple patient journeys for presumably frail older people with acute respiratory infections (including community-acquired pneumonia (CAP)). A secondary aim was to explore diagnostic and treatment strategies that could potentially contribute to the organization of pathways, including a hospital at home patient journey. Using design thinking methodology we believe we can reach these aims by involving the patients who are eventually receiving a new way of care delivery [30].

## Developing the ICP

### Setting

The region for which this ICP was created is an urban area in the Netherlands. This region has a total population of 1.122.240 inhabitants [31]. More than 28% of the regional population is 60 years and older, and more than half of the people who are 65 years and older have a chronic disease [32]. In the Netherlands, everyone has a GP and when indicated, people are referred by their GP to the hospital.

### Design and participants

‘Design thinking’ methodology was used in the current project to drive the innovation process up to the ‘testing and implementation’ phase. Design thinking is a systematic innovation approach that has deep empathy for the desires and needs of end-users (in this case older patients with an acute moderate-severe respiratory infection or CAP and their caregivers) [30,33]. We used design thinking because the end-users are involved from the beginning and patient (and patient representatives) and provider involvement is in our opinion a prerequisite for developing care for their medical needs.

**Figure 1:**
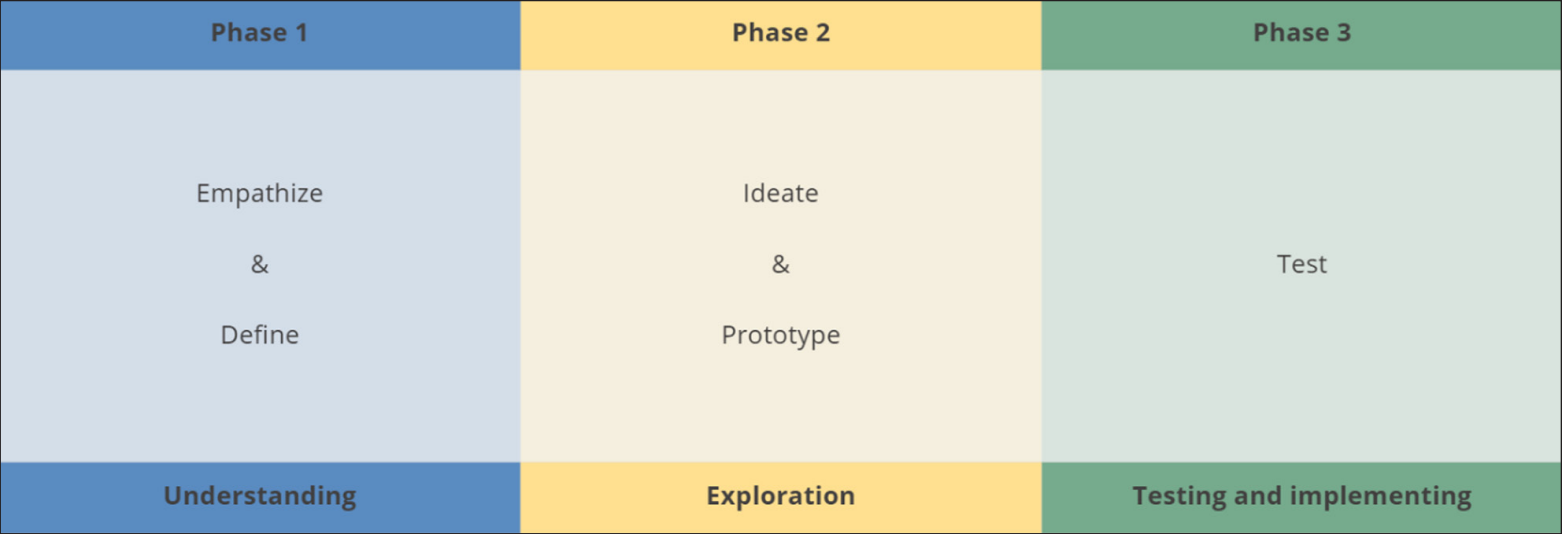
Phases and steps of design thinking.

The design thinking method we used has three phases, and a total of five steps, see Figure 1. The first phase is the understanding phase, with steps 1) Empathize (the needs of end-users) and 2) Define (what is the problem), the second phase is the exploration phase with steps 3) Ideate (brainstorm for solutions) and 4) Prototype (rapid prototyping of the solution). The last phase is the testing and implementation phase with step 5) Test (test the prototype). This is followed by the implementation of the program, which is currently being planned and is therefore outside the scope of the current paper.

### Co-creation of multiple patient journeys

**Figure 2:**
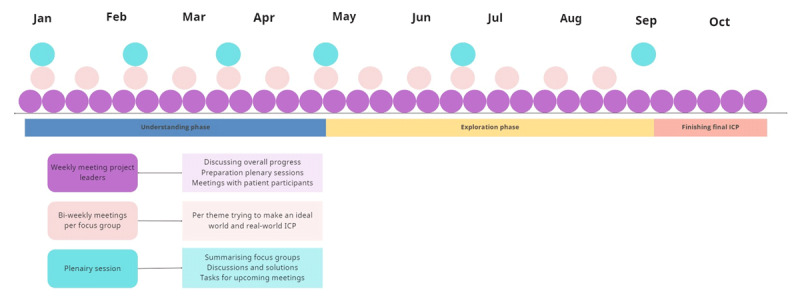
Schematic time display with meetings and activity examples. With the design thinking phases below. ICP: integrated care pathway.

Due to COVID-19 restrictions during the study period, every session was held online lasting one to two hours. At the first plenary session, participants were randomly assigned into groups and questioned about what important topics were to consider regarding respiratory disease treatment. Important topics that were apparent were: 1) diagnostic testing, 2) treatment, 3) monitoring, and 4) communication. All plenary sessions were chaired by the project leaders (RP, IK, CvN). Participants were recruited through their own network and local care organisations. When during the sessions expertise was lacking, a new expert was approached and invited to participate. As such, a medical microbiologist, a second radiologist, and a hospital manager were added. At the end of the first plenary session, all participants were assigned to one of four expert focus groups, based on their expertise (See appendix 1 for focus group allocation and background). The groups’ themes were the same as the main topics: 1) diagnostic testing, 2) treatment, 3) monitoring and 4) communication. Meeting bi-weekly per theme group and every six weeks in plenary session, together these focus group sessions were considered one cycle of our design thinking method, see Figure 2 for a schematic time display.

Throughout our design thinking co-creation sessions a total of 23 stakeholders participated. This included patient representatives, GPs, specialists in elderly care medicine (ECM), managers involved in acute care and nursing home admittance (from the two largest regional organisations), medical specialists and residents representing internal medicine, pulmonology, radiology, clinical chemistry, hospital pharmacy, and microbiology.

For co-creating the various patient journeys, we followed the first two phases of design thinking: 1. the understanding phase, with empathizing and defining, and 2. the exploration phase, with ideating and prototyping (Figure 1).

### 1. Understanding phase: Creating the patients’ ideal world

During this first phase, the focus of the group sessions was to empathize with the experiences of our patient participants. This was discussed during the first plenary session including topics such as ‘what are their wishes when it comes to the four topics?’ and ‘what are the problems patients (and their doctors) do foresee?’ Using the steps of empathizing and defining to discover the needs of patients and define the problems they encounter. The focus was to define patient journeys in an ‘ideal world’ from the patient’s perspective taking the four topics into account. In this phase during plenary sessions every focus group introduced their specific discussions, considerations and questions until a consensus was reached. After three cycles of six weeks, the optimal patient journeys (in the ‘ideal world’) were defined on the needs of the patients.

### 2. Exploration phase: Real-world implementation solutions

During the exploration phase, the goal was to further develop (ideate) the patients’ journeys based on the real-world context and provide a prototype. The four focus groups were disbanded and a new focus group with already involved stakeholders and patient representatives was formed. They were tasked to address anticipated barriers for implementation in the real-world situation. This focus group also met bi-weekly and brainstormed for practical solutions. They also discussed their findings during plenary sessions with all involved stakeholders. This also took three cycles of design thinking. A final conceptual version of the ICP consisting of multiple patient journeys was formed and distributed by all stakeholders to their peers in the region to gather feedback and advice on the feasibility and usefulness in local routine care. All stakeholders reached at least two or more peers. All feedback received from these peers, was then discussed and incorporated during the final plenary session, after which a definitive ICP was drafted. Changes made, for example, were creating new phone numbers for the acute care team, pharmacy agreements on delivering medication to the patient’s homes, and changes in the explanatory patient/caregiver forms for at-home monitoring.

### Patient participants

Two patient representatives participated in all plenary sessions. The first is a patient expert and board member of the national society of patients with pulmonary diseases. The second patient representative is a board member of the regional elderly council who is also an experienced caregiver. They speak on behalf of their members and from own experiences. Before a plenary session was held, the project leaders (RP, IK and CvN) informed them of the latest developments from the bi-weekly sessions so that they could prepare questions and fully interact in the discussions. As such, from a patient perspective, together we were able to develop an acceptable, realistic, and feasible hospital at home track, and other patient journeys.

### Ethics

For developing the ICP, it was not contributing to obtaining ethical approval from a medical ethics committee.

## Results

**Figure 3:**
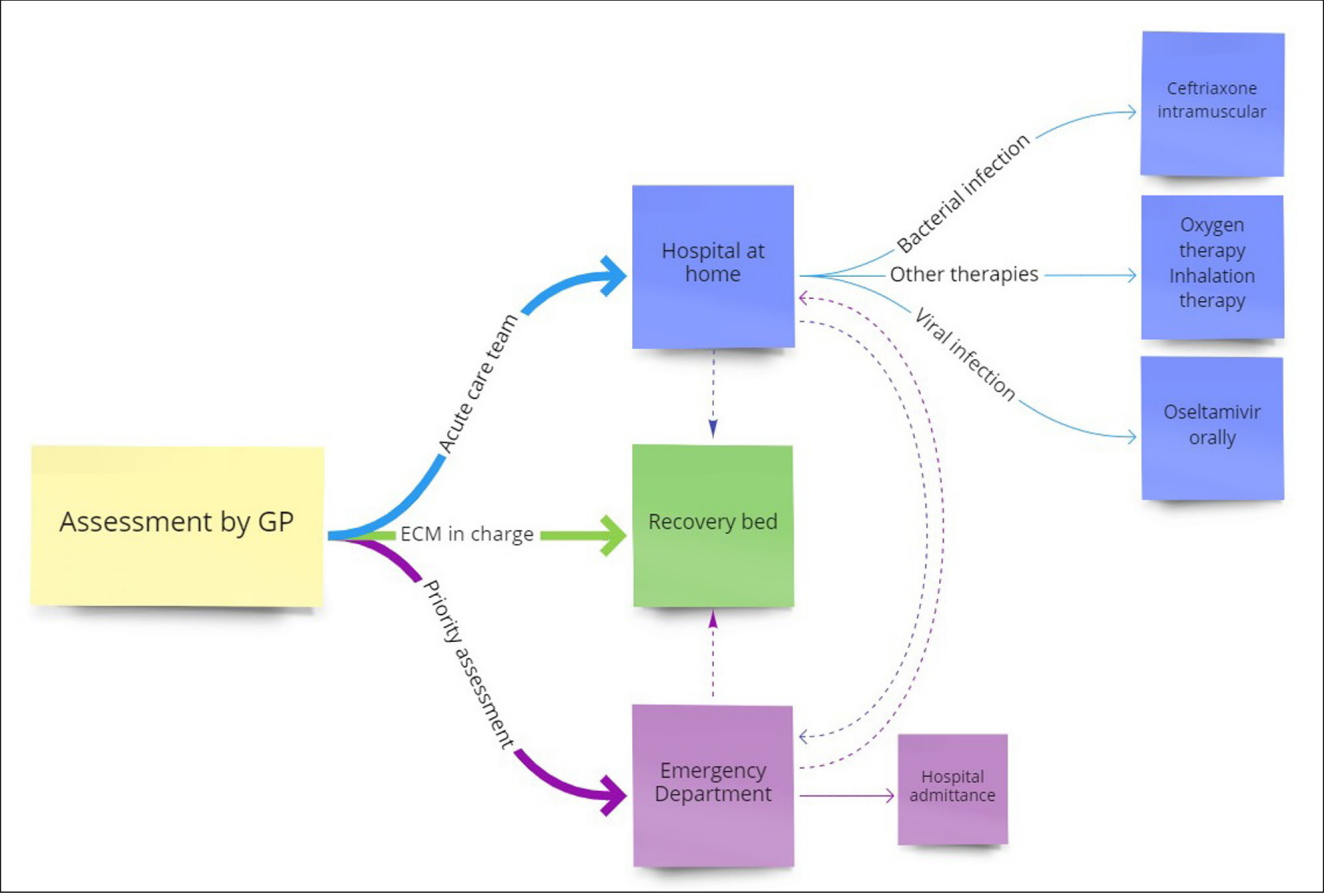
The ICP includes three possible patient journeys. The acute care team consists of a daily visiting nurse, the responsible home care organisation and an emergency call centre for the patient and their caregiver. Thicker lines indicate the three described journeys. The dotted lines showing optional redirections. GP: general practitioner, ECM: specialist elderly care medicine.

The definitive ICP consisted of three embedded patient journeys, including a hospital at home journey. (Figure 3) During co-creating sessions for the ‘ideal world’, four patient journeys were drafted, consisting of 1) Hospital at home; 2) Tailored visit to the ED; 3) Admittance to a readily available recovery bed, and 4) Side-stepping the ED. However, during the exploration phase only the first three could be included in the definitive ICP; see the discussion below and Figure 3. Each of the three patient journeys began by following the steps developed by the diagnostic testing and treatment focus groups. Additionally, we established new regional collaboration agreements for consultation between GPs and ECM specialists, internists, and pulmonologists.

### Patient journey 1: Hospital at home

In the first journey, treatment in the home setting is key. This is the pathway that most closely reflects the patients’ ‘ideal world’, with the GP leading this pathway from beginning to end. After an initial assessment establishes clinical suspicion of moderate to severe pneumonia, the ‘diagnostic testing phase’ begins. In the ‘ideal world’ situation, point-of-care testing (POCT) of kidney function would take place based on analysis of creatinine, electrolytes such as sodium and potassium, and C-reactive protein as an inflammation parameter, in addition to microbiological nasopharynx swab tests for influenza and the current SARS-CoV-2. Although considered a major advantage by patient representatives, the solutions suggested by the diagnostic testing focus group for the proposed new regional ICP pathway are not yet available for home implementation. Blood tests and a nasopharyngeal swab for respiratory pathogen detection are normally ordered by a GP and performed in regional laboratories. To ensure a patient is sufficiently well to be treated at home, an assessment for delirium risk is conducted based on current guidelines [34]. Furthermore, an adjusted validated “Acute Presenting Elderly Patient” (APOP) questionnaire is used to derive an assessment for frailty, which is then compared to the most recent UPRIM Frailty Index score derived from electronic medical records [16,35].

Once a clinical diagnosis of pneumonia has been confirmed, the GP can treat patients at home with four additional medical options beyond current guidelines of respiratory tract infection in primary care:

1. The first option is to prescribe a guideline-recommended cephalosporin (ceftriaxone), administered by single daily intramuscular injection for five days. This is especially suitable for patients suffering from nausea or who might not be able to swallow oral antibiotics.
2. The second option is to prescribe oseltamivir or baloxavir, a neuraminidase inhibitor, in case of a suspected or confirmed influenza infection. Currently, this treatment is restricted to hospital care in the Netherlands, and as the medication is not recommended in the Dutch GP guideline on respiratory infections it will not be reimbursed by health insurance companies when prescribed in primary care [36]. When COVID-19 is suspected or established, the latest guidelines apply.
3. The third option – a lesson learned during the COVID-19 pandemic – is treatment with low rates of home oxygen therapy at home under the guidance and initiation of the GP.
4. There is a final additional option for patients with signs of concurrent obstructive airway disease; the GP can treat with inhalation medication and/or oral corticosteroids after consultation with a pulmonologist.

The first three treatment options would be especially suitable on a patient-by-patient basis if diagnosis was possible based on additional on-site examination and POCT. These additional treatment options are of major potential benefit to the patients and their informal caregivers.

To monitor patients in their home setting, agreements on inclusion criteria were first reached (Table 1).

**Table 1:** Inclusion criteria for hospital at home journey.


The patient has a (suspected) respiratory infection

Patient and caregiver are motivated and able to learn monitoring skills*

Patient and caregiver are able to use measuring devices to measure vital values

Home care is already sufficient or initiated by the GP

A concise individual care plan has been formulated by the GP and patient

Oxygen saturation SpO2 =/> 92%, with a maximum of 5 litres oxygen suppletion and a breathing rate =/< 24 per minute**


* Assessed by own GP. ** Or adjusted values relevant to the individual patient.

**Figure 4:**
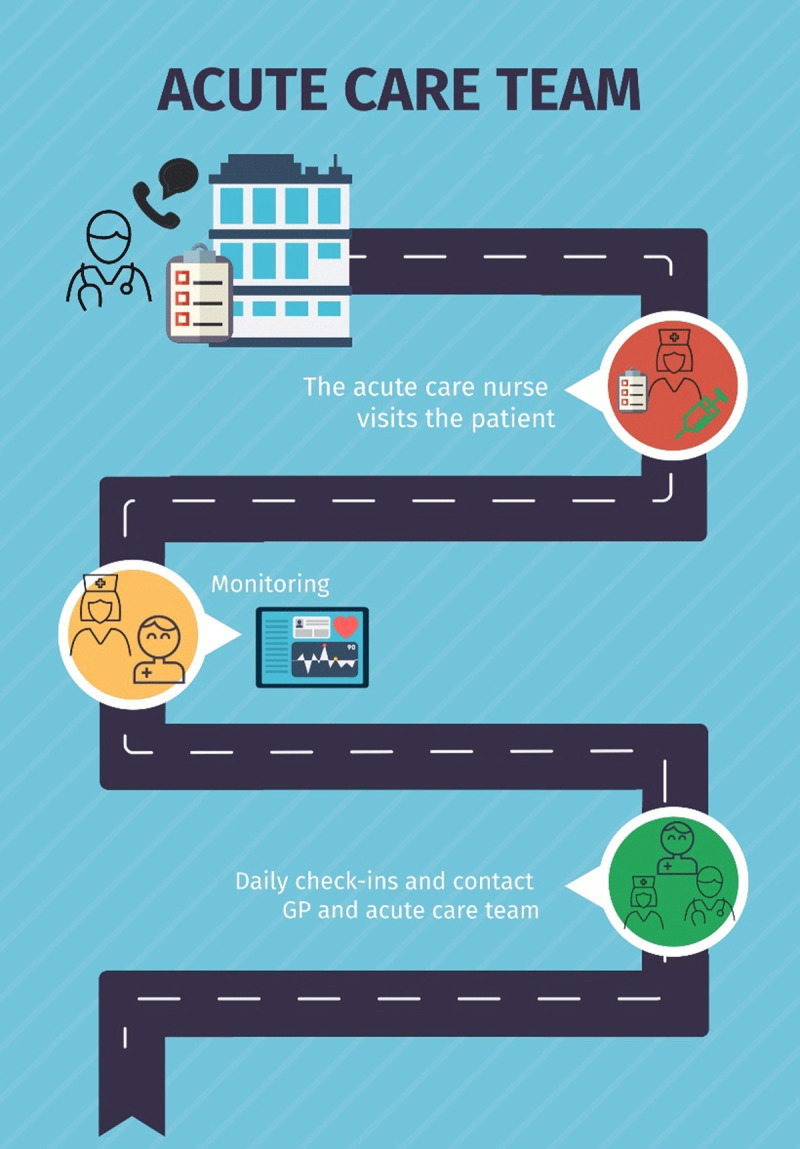
Hospital at home journey, acute home care team.

The GP formulates a treatment plan and an individual care plan together with the patient, and/or with the caregiver, which is then shared with the acute home care team (Figure 4). An important prerequisite here is that an acute home care team already exists in our region. The responsible pharmacies deliver the antibiotics and/or oseltamivir/baloxavir to the acute home care team’s main office. An acute care nurse subsequently visits the patient within four hours to administer the treatment and deliver a monitoring kit. This kit includes a saturation device and a thermometer and will be used to measure vital values. These values, together with breathing rate and heart frequency, will be daily monitored by the acute care team nurses and GP. Daily (online when possible) contact moments between the patient/caregiver and acute care team and or GP are organized for the following three to five days (Figure 4). Although a regional digital communication application that includes patients would be highly desirable, this is not currently available.

### Patient journey 2: Tailored visit to the Emergency Department

The second patient journey involves a tailored visit to the ED. This journey will be chosen if, after an initial assessment by the GP and consultation with a hospital specialist, an extended medical assessment is considered necessary. A special referral template is developed and can be used to refer to the hospital according to the ICP agreements. A priority assessment will be pursued to minimize time spent at the ED and will be performed according to the standard of care. Within this patient journey, the hospital specialist takes the lead and decides if hospital admittance is necessary. However, when medically possible, treatment at home is still the preferred setting. If the patient returns home after the ED visit, prescriptions and an individual care plan will be organised by the hospital specialist. The hospital specialist and GP will establish clear follow-up agreements, and the GP will again take the lead in the home setting. The hospital specialist also has the option of referring to a readily available bed in a home care facility under the supervision of an ECM (Figure 3).

### Patient journey 3: Admittance to a readily available recovery bed

The third co-created patient journey is suitable when treatment in a hospital at home setting is not considered possible and hospital admittance is not necessary. In this journey, responsibility for care can be directly transferred from the GP to the ECM at the nursing home care facility. The two largest regional home care agents involved have arranged a guaranteed option on a so-called recovery bed for patients included in this ICP. This journey is thus available after home assessment by the GP or as a result of an ED visit. (Figure 3)

### An additional ideal world patient journey: Side-stepping the Emergency Department

This ‘ideal world’ patient journey was seen as very valuable by both patient representatives and GPs. The aim of this journey was to avoid admittance to the ED by organizing additional, tailored medical assessments by the Radiology and Chemistry departments. For example, when a GP requests blood tests, a microbiological nasopharyngeal swab and a chest X-ray, this patient journey would allow these without admittance to the ED. This ensures that a patient will avoid having to spend hours at the ED for these tests. This fourth patient journey unfortunately encountered so many real-world barriers (primarily logistical and privacy law-related) – most without ready solutions – that it was impossible to include it in the definitive ICP. See also Table 2.

**Table 2:** Example barriers encountered per patient journey.


PATIENT JOURNEY	PRACTICAL BARRIERS	BARRIERS REGARDING COMMUNICATION

1. Hospital at Home	POCT at home by GP, financial health insurer means for oseltamivir at home, language barriers in our urban region.	Communication application between patients and healthcare professionals.

2. Tailored visit to the Emergency Department	Waiting times at the ED, using the new referral template by GPs, same workflow at two different hospitals.	Communication between hospital specialist and GP regarding discharge to home after office hours.

3. Admittance to a readily available recovery bed	After hours admittances at the nursing homes, transportation waiting times, financial barriers for the availability of a recovery bed.	Involving all ECMs in the region to adhere workflows, even when not working for the involved nursing homes.

4. Side-stepping the Emergency Department	Waiting time for the ambulance at the hospital, legal responsibility for the patient when in the hospital, transportation to radiology and chemistry department.	Communicating results from radiology or chemistry lab to GP.


## Discussion

In this study we aimed to use design thinking to co-create a regional, multidisciplinary ICP consisting of multiple patient journeys for community-dwelling, presumably frail older people with an acute respiratory infection (including CAP). The involvement of patient representatives during all developmental stages differentiates the ICP described here from most other ICPs. Four patient journeys were explored and three were further designed.

### Designing hospital at home

One of the clear strengths of our ICP is the hospital at home track in the first patient journey. We believe this may have a major impact on older patients with an acute moderate-to-severe respiratory infection in our region. Patients and their caregivers stay more in control, have another possibility of treatment to consider, and have the possibility to stay at home. The original hospital at home concept includes specialist secondary care for specific patient groups that would normally receive treatment in a hospital but opted to receive treatment at home, with the support of their respective families [37]. The hospital at home concept is continually adapting and now ranges from complete home treatment and care, early hospital discharge to home, to hospital at home schemes that support admission avoidance [21,37,38]. Our ICP is closest to the latest concept, with appropriate care at home (or at a nursing home) allowing hospital admission to be avoided.

When facing current and future demographic and organisational challenges such as an aging population and increasing pressure on healthcare resources, a hospital at home care pathway may offer one solution. Treatment in the home setting is a common and frequently chosen option for multiple medical conditions, including treatment of pneumonia. Reviews by Shepperd et al. and Gonsalves et al. found no differences regarding mortality and readmission for home-based versus in-hospital treatment [37,38]. Furthermore, home-based treatments have been evaluated and confirmed to be safe [39,40,41,42]. Nevertheless, studies concerning patient preferences and satisfaction have shown mixed results, with patients favouring care at home in some studies but hospital-based care in others [43,44,45]. In contrast to our approach, these studies did not involve end-users during the development phase, evaluating hospital at home tracks for feasibility and end-user acceptance only after implementation. If older patients do not need or want an intensive care trajectory, treatment at home becomes an appropriate option given a well-organized ICP. Patients have an overall preference for being treated at home, even in cases with a high risk of an unfavourable outcome. [46,47,48] In our opinion taking patient representative’s opinions into account at an early stage, ensuring equal participation of all stakeholders in co-creation sessions and showing commitment to a partnership contract are all critical factors for the successful development of an ICP. Equal participation was achieved by making people responsible for ownership, collecting discussion points in advance of the bi-weekly and plenary sessions and giving everyone the opportunity to interact in the discussions. An ICP must include teamwork, information exchange, local guidelines and new treatment options.

### Design thinking in healthcare

Including patients, patient representatives and caregivers in the development of transitions of care is becoming more common [30,33,49,50]. Not just with design thinking but also other forms of participatory approaches exist, nationally and internationally [51,52]. Seidel et al. [49] concluded that new multidisciplinary teams, like ours, working on innovations have a greater potential benefit if they use a design thinking approach. Especially for the need-finding and the prototyping, we also have this experience with the input of our patient representatives. For example, challenges we faced were the “problem-solving-attitude” of the medical healthcare professionals during the first phase of just listening to the patient’s ideas about their ‘ideal world’. According to Roberts et al. [30] design thinking works best when it is used in the beginning of the innovation process. Sometimes problems are not yet clear, but the end-goal is. During this co-creation process, it becomes clear what the needs of end-users are and what problems they encountered. Integration of multiple disciplines is a result of design thinking, not only in research but also in practice, according to the historical review of Auernhammer et al. [50] With our approach and involving different stakeholders, we reached to have multiple disciplines integrated in care delivery in our region. In retrospect, we missed the health care insurer to be involved during this process.

### Designing integrated care pathways

Despite the best intentions, there is much debate about the effectiveness of ICPs in practice [28,29,53,54,55]. While a modest impact can be expected on clinical outcomes, ICPs have a substantial impact on the processes of care delivery. Although achieving only a modest clinical impact, most ICP interventions actually increase costs (mostly in the short term) due to ICP implementation costs [54]. A review by Allen et al. of determinants associated with effective ICPs, found that only relatively predictable trajectories of care seem to benefit from ICPs, presumably by supporting active care management and delivering relevant clinical interventions [53]. Schrijvers et al. showed other benefits of effective ICPs including promoting guideline adherence, improving documentation on treatment goals, as well as improved communication between patients, their caregivers and health care professionals [55]. Based on these arguments of Allen et al. and Schrijvers et al., our ICP contained sufficient necessary characteristics such as clinical interventions, documentation on treatment goals and the relative predictable trajectory.

ICPs have been shown to be ineffective when the main goals are efficiency gains across variable trajectories, service quality improvement or overall cost reduction. The ICP developed by us was designed to boost the quality of care across the primary and secondary care interface for a specific group of patients at risk of medical deterioration. Our way of designing an ICP suggests that trying to change current local care is possible when health care providers have an intrinsic incentive and are enthusiastic and motivated. To establish a multidisciplinary care approach that addresses an existing complex care need, such as treating older people at home, the collaboration, participation and commitment of all stakeholders from the various levels of regional care is an absolute requirement. We felt we achieved this through partnership contracts, equal participation and having the same goal. Moreover, when successful in real practice, this ICP might be used as an example that could be used for other common acute infectious diseases in older adults, like complicated urinary tract infection or skin infections.

Creating and implementing an ICP for an intervention that seeks to change entrenched habits in healthcare will naturally encounter obstacles. Recently, Seckler et al. reviewed studies on potential facilitators and barriers to the implementation of a new ICP for multidisciplinary care at the primary-secondary care interface [29]. As the review focused on older patients in an outpatient setting or a hospital stay of under than 24 hours, the lessons learned may well be applicable to the implementation of our ICP. Identified barriers included multi-morbidity among older patients, lack of incentives for providers and expectations that ICPs would be time-consuming. In our peer feedback, some of these barriers were mentioned, especially the time-consuming and lack of incentive. By co-designing and co-creating the patient journeys in our ICP, we have tried to overcome these potential barriers to implementation.

## Strengths and limitations

The major strengths of our study included the involvement of patient representatives during the entire process and the creation of an ‘ideal world’ care process. The preparations of patient representatives for the plenary sessions provided us with valuable input and they remained critical throughout the entire process. We could therefore create the practical patient journeys from a patient’s perspective, while keeping logistical boundaries in mind. This was very helpful in our used design thinking method, which is another strength. In addition, we were able to combine new treatment options into one ICP, including a hospital at home track, tailored to our regional and local capabilities but with the potential for wider dissemination. The variety and effective engagement of stakeholders is especially important in an urban setting where large home care organizations are more common. Other strengths of our ICP are the fact that little GP time is consumed when including patients, and the intervention is relatively simple and well organized. A limitation of the study was the inability to overcome all barriers encountered during the co-creation of patient journeys. For example, a medical communication tool that would have allowed patients and health care professionals to communicate and monitor health conditions could not be included due to potential privacy issues. A second limitation was that recovery beds from other regional organizations could not be used, despite the involvement of the two largest home care organizations. Another limitation was the rather limited number of patients involved in our design thinking method. Finally, financial barriers were not addressed during the co-creation of our ICP, a nonetheless important prerequisite for ICP implementation.

## Lessons learned and recommendations

### Practice

- Design thinking is a suitable method to develop care pathways as it promotes a multidisciplinary approach and integration of care.
- Taking patients’ perspectives for their ideal setting as starting point takes the development to a higher level, with doctors sometimes having no clue about what is important to patients, and it keeps medical professionals humble and realistic.
- New forms of local cooperation emerge through ICPs.

### Policy

- Local stakeholders are necessary for local practical workflows.
- Taking other lessons learned (during a pandemic) into new local practice and making them standard-of-care.
- Barriers in hospital regulations are sometimes unsolvable to transform the ‘ideal world’ into the real-world.
- Invite the healthcare insurer to the designing table and foresee what financial barriers there will be in the new system.

## Further study

The implication of our design thinking approach with the aim to design an ICP is a feasible hospital at home treatment option. This ICP is a change in bringing treatments closer to the patients. The hospital at home treatment option is expected to highly contribute to a positive patient experience, decreasing costs and better care for this older patient population. The ICP is ready for iterative implementation in our region in the fall of 2022. We heed the call of Seckler et al. [29] to research the implementation of a new developed ICP and evaluate the process and outcomes. The different patient journeys will be assessed and adjusted if necessary and the hospital at home treatment will be monitored closely.

## Conclusion

In this study we developed an ICP for community-dwelling presumably frail older people with an acute respiratory infection. Using a design thinking method to discover the local possibilities is practical and helps to find and develop new options to deliver care the way a patient wants to receive it. For this latter it is of tremendous value to include patients and their representatives, not just including them at the end of a development process but from the start. Taking the ‘ideal world’ to the real-world comes with challenges, but we believe that in time all barriers will be solved. In our region, a prerequisite for the hospital at home journey was the existing acute care team. They are, together with the GP, the focal point in this treatment option. Three patient journeys were co-created: treating older people with respiratory infections in a hospital at home journey; a tailored visit to the ED, which has the primary benefit of allowing fast, focused diagnostic assessment in an otherwise time-consuming and stressful medical environment; a readily available recovery bed for those patients for whom treatment in a hospital at home setting is not possible and hospital admittance is unnecessary. The ICP co-created in this study, comprising three local, practical patient journeys, will be implemented and evaluated in the near future.

## Additional File

The additional file for this article can be found as follows:

10.5334/ijic.6991.s1 Appendix. Focus group allocation.
