# Supplementary material for: Using Design Thinking for Co-Creating an Integrated Care Pathway Including Hospital at Home for Older Adults with an Acute Moderate-Severe Respiratory Infection in the Netherlands
Source: Int J Integr Care. 2023 Jun 21;23(2):30. doi: 10.5334/ijic.6991 (PMC10289046; doi:10.5334/ijic.6991)
Supplement: Appendix. — Focus group allocation. [file ijic-23-2-6991-s1.pdf]

Focus group allocation

Diagnostic testing

- Professor, general practitioner in primary care practice and public health
- Internal medicine resident and PhD candidate, chair
- Internal medicine resident
- Internal medicine specialist
- Clinical chemist
- Elderly care physician
- Radiologists
- Medical microbiologists

Treatment

- Elderly care physician resident, chair
- Elderly care physician
- Infectious diseases specialist
- Hospital Pharmacist
- Pulmonologist
- Professor infectious diseases
- Manager acute care at home

Monitoring

- Primary care physician, chair
- Primary care physician
- Manager acute care at home
- Manager elderly care and housing
- Elderly specialists

Communication

- Elderly care physician, chair
- Primary care physician
- Managers home care and housing

Real-world group during exploration phase

- Internal medicine specialists and resident
- Pulmonologist
- Representatives home care agents
- Clinical chemist
- Hospital pharmacist
- Medical microbiologist
- Primary care physicians
- Radiologists
- Elderly care physician
